# Evaluation of Home-Based Rehabilitation Sensing Systems with Respect to Standardised Clinical Tests

**DOI:** 10.3390/s20010026

**Published:** 2019-12-19

**Authors:** Ioannis Vourganas, Vladimir Stankovic, Lina Stankovic, Anna Lito Michala

**Affiliations:** 1 Department of Electronic and Electrical Engineering, University of Strathclyde, Glasgow G1 1XQ, UK; vladimir.stankovic@strath.ac.uk (V.S.); lina.stankovic@strath.ac.uk (L.S.); 2 School of Computing Science, University of Glasgow, Glasgow G12 8QQ, UK; annalito.michala@glasgow.ac.uk

**Keywords:** automated timed up and go test, automated five time sit to stand test, self-evaluation, evaluation of sensor systems, non-intrusive sensing, sensing for health

## Abstract

With increased demand for tele-rehabilitation, many autonomous home-based rehabilitation systems have appeared recently. Many of these systems, however, suffer from lack of patient acceptance and engagement or fail to provide satisfactory accuracy; both are needed for appropriate diagnostics. This paper first provides a detailed discussion of current sensor-based home-based rehabilitation systems with respect to four recently established criteria for wide acceptance and long engagement. A methodological procedure is then proposed for the evaluation of accuracy of portable sensing home-based rehabilitation systems, in line with medically-approved tests and recommendations. For experiments, we deploy an in-house low-cost sensing system meeting the four criteria of acceptance to demonstrate the effectiveness of the proposed evaluation methodology. We observe that the deployed sensor system has limitations in sensing fast movement. Indicators of enhanced motivation and engagement are recorded through the questionnaire responses with more than 83% of the respondents supporting the system’s motivation and engagement enhancement. The evaluation results demonstrate that the deployed system is fit for purpose with statistically significant (ϱc>0.99, $R^{2}>0.94$, ICC>0.96) and unbiased correlation to the golden standard.

## 1. Introduction

In an era when more and more patients are required to perform rehabilitation activities in their own environment, there is a need for systems suitable for home use. Since the patients are meant to interact with these systems alone, without support from a specialist, acceptance and lasting engagement are crucial. In [1] four criteria are established that a successful home-based rehabilitation system should fulfil to lead to wide acceptance and long engagement, including the need for non-intrusiveness (e.g., no cameras), absence of any wearable component, ease of use and affordability.

However, home-based rehabilitation equipment that fulfils the aforementioned criteria usually cannot meet the specifications of clinical rehabilitation systems. Therefore, home-based rehabilitation equipment must be rigorously evaluated against specific and measurable medical tests [2] in order to meet medical standards. These tests combine multiple daily activities such as walking, sitting and standing.

A detailed literature search performed this year [1], reaffirms the previous findings of [3] that existing automated self-evaluation systems do not meet the above identified criteria, particularly in terms of acceptance and low-cost requirements. This paper takes further the findings of [1,3], and proposes practical methodological steps for evaluating new home-based rehabilitation systems in terms of meeting the medical specifications and the four acceptance criteria. To demonstrate the proposed evaluation methodology, we evaluated a home-based rehabilitation system that satisfies the above four criteria of patient acceptance, and evaluate its performance against medically accepted standard tests, which are discussed next.

### 1.1. Patient Evaluation Tests

Different tests exist for the evaluation of gait, balance and mobility of subjects. These tests are used to measure/evaluate specific characteristics relevant to the subject’s clinical condition. The outcomes of these tests help identify underlying illnesses or support recovery after an illness has occurred (e.g., post stroke) [4]. Most well-known and used tests are presented in Table 1. The functional reach test would require a combination of several sensor systems, including wearable sensors to capture vestibular motion. Berg balance scale, performance oriented mobility assessment, and balance evaluation system tests all assess static balance and posture, but require wearable or intrusive sensing techniques as well as a specialist being present during the tests. Hence, the aforementioned tests are not suitable for self-assessment and home rehabilitation, where specialists may not be present. Falls efficacy scale and balance confidence scale self-evaluation are carried out via a questionnaire to describe daily activities. To automate and monitor all the activities covered in the questionnaire, the system cost would increase significantly. The balance error scoring system (BESS) test is targeting the younger segment of adult population and particularly athletes, with tasks that could be challenging for the less mobile. Finally, the timed up and go (TUG) and five times sit to stand FTSTS tests can be characterised by their simplicity, accuracy and suitability for all adults. Furthermore, TUG and FTSTS cover multiple activities with one test, and can be monitored by systems meeting the four criteria of patient acceptance, both in home and clinical environments.

Due to a variety of reasons, including socioeconomic [5], automated solutions for some of these tests have appeared. A significant motivator for the automation of these tests is the elimination of human error [3,6,7,8]. Indeed, in the majority of non-automated tests, the time is measured using a stopwatch, which inherently incurs human error [9].

The TUG test can be carried out in the home environment with a non-clinical person’s assistance. The only tool needed is a stopwatch to measure the time to complete the test [17]. The test algorithm is relatively simple, combines more than one daily activity and contains performance thresholds, as defined by the National Health Service (NHS) of the UK. For example, completion time exceeding 15 s identifies a patient at risk of falling [17,18].

However, factors such as age, gender, different levels of impairment or other medical conditions, can affect the accuracy of this assessment. Thus, different thresholds have been proposed to incorporate these factors, as presented in a study of 2084 subjects in [19].

To investigate, validate and evaluate the transferability of the automated sensor system, a second test was selected. The FTSTS test also incorporates basic motion linked to daily activities [20], but it complements TUG in assessing strength of lower limbs and durability, is time based and identifies fall risk [21]. Both TUG and FTSTS tests are approved by the NHS.

### 1.2. Contributions

In this paper we propose a generic methodology for evaluating a low-cost, non-intrusive, non-wearable, home-based rehabilitation system. This is evaluated in terms of accuracy, complexity and transferability to a variety of daily activities and other prognostic and diagnostic applications. The contributions of this paper are:

1. Deployment of a low-cost system to automatically perform the TUG and FTSTS medical tests.
2. A detailed methodology to assess a home-based rehabilitation system’s accuracy against the test specifications, benchmarked against NHS standard practice and ground truth established through video recording.
3. Demonstration of transferability to other daily activities and more than one NHS test.

The remainder of this paper is organized as follows. Section 2 presents related work in the field of TUG and FTSTS test automation. The methods proposed in this paper and experiments performed are presented in Section 3 and Section 4 respectively. Results are presented in Section 5 and discussed in Section 6. Finally, the conclusions of this paper are presented in Section 7.

## 2. Portable Sensing Tele-Rehabilitation Systems

With an acknowledged need for automating the TUG and FTSTS tests [5], several sensor-based systems have been proposed in the literature. These are reviewed based on the type of sensors used.

### 2.1. Camera and Video Systems

The state-of-the-art TUG test has a significant focus on intrusive solutions using cameras. A bidirectional communication video system has been proposed so that two specialists can assess one subject [22], and disabilities are simulated so that a wide variety of results can be attained. Other approaches utilise multiple cameras to identify risk of fall as well as the TUG completion time [23], and analyse each phase of the test [24].

More recent approaches improve the accuracy of early approaches [24,25] and utilise more advanced webcam sensors such as Kinect [6,25]. These provide better skeleton tracking and are suitable for home use as well as risk-to-fall categorisation [5]. For the FTSTS test, a CCD webcam approach is presented in [26] while in [27] Kinect is used with feedback provided in virtual reality. The latter was validated against both stopwatch and video recordings of the experiments. A combination of Kinect, pressure sensors and wearable components is investigated in [28].

All these approaches, however, have several constraints affecting both the accuracy and acceptance; namely camera positioning, people interference, lighting issues, floor quality for the Kinect sensors and most importantly, privacy issues.

### 2.2. Wearable Sensor Systems

Wearable sensors such as gyroscopes or accelerometers have also been heavily investigated. In [29], a combination of gyroscopes and accelerometer have been used to measure a combination of parameters such as angular velocity, step time average and maximum, and number of steps for patients with Parkinson’s disease. A fully automated two shank tri-axial accelerometer approach was able to capture additional gait parameters in [30]. Another approach [31] utilises two inertia measurement unit sensors placed on the lower back and leg, identifying hemiplegic subjects; however supervision is necessary. A single back loco Metrix triaxial accelerometer was proposed in [32] for Alzheimer’s patients and mild cognitive impairment subjects. A combination of wearable inertia measurement units with pressure sensors embedded in a chair is suggested [33] for patients with Parkinson’s disease.

In [34], a waist worn tri-axial accelerometer and a web interface application have been proposed for fall prevention measuring TUG, FTSTS and other tests; these are then remotely assessed by a specialist. For the FTSTS test, a 3D accelerometer placed on the hip, a 3D Gyroscope and a 3D magnetometer are proposed in [35], while an MTX 9 micro-electro-mechanical sensor tracker placed on the lower back was presented in [36]. An inertial sensor system, with three lower limb and two upper limb sensors, combined with a Nintendo Wii pressure sensor platform were proposed in [37]. On the other hand, refs. [38,39] propose wearable pressure sensors on the subject’s feet.

Mobile phones with accelerometers have also been used as wearable sensors. In [40,41,42], an android phone was placed on a subject’s back, while in [43], it was placed on the waist for the TUG test for different medical conditions. Similarly, a self-evaluation app is presented in [44].

These approaches might be easy to use by tech-savvy and healthy subjects. However, acceptance and engagement in the luddite/non-tech-savvy is low due to a variety of reasons: requirement of specialists to place the sensors, complexity of displayed information, and lack of familiarity with technology [1].

### 2.3. Other Sensor Systems

Alternative approaches have been proposed that automate tests in the home environment without the use of intrusive or wearable sensors. In [45], pressure sensors are installed in living room furniture for the TUG test, but have so far failed to produce the required accuracy. Other systems have specifically focused on capturing gait speed but not as part of the TUG test [46,47]. High accuracy is achieved in [48] where seven sensors are used for TUG, combining light barrier sensors, pressure sensors, a laser scanner as well as long cables and a wearable pair of white cuffs, but feedback to users was not supplied.

Several systems have been developed to identify sit or stand in this category [49], mostly focusing on the use of pressure sensors [50] but have not automated the FTSTS test. Such systems appear to be an attractive solution given that they are not intrusive, could provide a solution tailored to the user needs, are extendable and integrated into daily environments such as homes [3,51].

## 3. Methodology

In this section, we describe our methodology to assess the accuracy of home-based rehabilitation systems for the TUG and FTSTS tests, as described in Table 1 and Section 1.1. For that purpose, we deployed a home-based rehabilitation sensor system, which satisfied the four criteria of [1], namely non-intrusiveness, did not contain any wearable component, was easy to use and low cost. The system comprised two time-synchronised blocks, each assembled using a micro-controller and a modified BISS0001 passive infrared (PIR) sensor to capture the time a subject took to walk between two points as he/she performed the TUG test (through horizontally spaced sensor blocks). Replacement of the electrical components as well as optimisation of the lens yielded sensitivity range of up to 1 m. And capture angle of 30°× 30°. The digital signal was adjusted to remain high after the trigger for a variable time between 0.25 s to 25 s, depending on subject speed. The FTSTS test was performed through a vertical arrangement of the two aforementioned sensor blocks, which measured the time from sit to stand. The sensor allowed for vertical and horizontal arrangement. The two sensor blocks were optimally placed for both tests to minimise false positives (i.e., when a motion that was not part of the test was picked up), and false negatives (i.e., when a motion that was part of the test was not picked up). Though applied to this system for demonstration purposes only, the proposed evaluation methodology is generic and can be followed for assessment of other similar home-based rehabilitation systems.

### 3.1. Participants

In total, eight healthy subjects were recruited to take part in the experiment in order to evaluate the proposed system for TUG and FTSTS tests. The subjects were recruited to illustrate the proposed methodology and to demonstrate if a system (any system) meets the desired requirements. The subjects were seven males and one female aged 22 to 41 (mean age $\bar{\chi }=30.25$). The mean height of the participants was $\bar{\chi }=1.74$ cm, with standard deviation of σ=0.12 and weight $\bar{\chi }=76.5$ kg with σ=12.14. This range of heights and weights allows for evaluation of the technology in a variety of scenarios even though it is predominantly representative of characteristics relevant to male adults [52,53,54].

The eight subjects were over the age of 18 with good vision (with or without corrective aids), they were given a participant information sheet explaining the procedure, and were able to provide consent. They followed instructions in English and attended a single appointment at the lab.

Exclusion criteria were used for subjects that were pregnant, had a hearing and/or visual problems that was not corrected, subjects that were unwell or were taking medication potentially compromising their ability of mild physical activity, subjects with significant speech problems affecting the safe execution of the experiment and subjects with vestibular impairments, heart or respiratory conditions that limited their ability to walk. The study was approved by the University of Strathclyde ethics committee and a data management plan for data security was in place.

Following a similar approach to [22], subjects were asked to simulate various disabilities. The simulated disabilities and number of repetitions are discussed in Section 4 for each test. The total number of individual experiments were 184 for TUG and 40 for FTSTS.

To demonstrate that the evaluated technology is able to record TUG and FTSTS results that are relevant to a wide range of adults, we compared the TUG and FTSTS completion time recorded during the simulations to the completion time reported for healthy and geriatric elderly (>65 years) and adults (>18 years) in the Shirley Ryan Ability Lab [53,54] international database. For TUG test, a total of 48 studies were analysed in the database of 6632 participants with a variety of conditions. Of those, only 10 studies report male/female populations; seven are predominately male at 67.3% on average, while three have male populations of 36.3% on average. For FTSTS test, a further 23 studies were analysed with a total of 7794 participants in the adult and elderly groups. Of those, only two studies report male/female populations at an average of 48.7% male predominance. A two-sample t-test analysis was performed with the hypothesis that recorded completion time distribution for each of the simulations was equal to completion time distribution reported in the database for conditions relevant to the simulated disabilities. The selected groups are presented in Section 5. As the female representation in the database is on average 49.23%, the hypothesis will also further support our experimental results not being overly biased towards male participants.

### 3.2. Statistical Data Analysis Measures

To identify agreement between the results obtained by the automated sensor system and the video measurements we carried out the Bland–Altman 95% bias analysis. This method is widely used in the medical field when comparing two measurements of the same variable. For each pair of measurements the x-axis illustrates the mean and the y-axis the difference. The method also provides the lower and upper level of agreement and establishes acceptable limits [55]. The percentage error (PE) of the measurements of the experiment is calculated following the Bland–Altman method based on the upper and lower limit of agreement (LOA) according to [56]:

(1) $$PE=\frac{Upper_{LOA}-Lower_{LOA}}{\bar{\chi }}$$

Lin’s concordance correlation coefficient (ϱc) (Equation (2)) was calculated to compare the proposed automated sensor system measurements against a “gold standard” or “ground truth” measurement as one of the most well-established methods to assess agreement [57], as follows:

(2) $$\varrho c=\frac{2*\rho *\sigma_{system}*\sigma_{video}}{({\bar{\chi }}_{system}-{\bar{\chi }}_{video}{)}^{2}+\sigma_{system}+\sigma_{video}},$$

where ρ is the correlation coefficient, $\sigma_{system}$ and $\sigma_{video}$ represent the standard deviation, of the automated sensor system and video system, respectively, and ${\bar{\chi }}_{system}$ and ${\bar{\chi }}_{video}$ are the mean of the automated sensor system and video system data points, respectively.

The intraclass correlation coefficient (ICC) was calculated as assessment of the reliability of the measurements [58]. The ICC was evaluated after conducting analysis of variance of two factors without replication. Finally, linear regressions analysis was performed to obtain accuracy, quantification and data trends.

## 4. Experiments

For each of the TUG and FTSTS tests that was conducted, data were recorded through:

1. a non-intrusive, non-wearable, low cost, motivation and engagement enhancing system that can be individualized, is simple and transferable;
2. a stopwatch following the instructions for specialists according to the NHS suggested method [17];
3. and a standard video camera as golden standard to avoid human error in the stopwatch method.

The automated sensor system is able to capture motion and time. It is a portable system, easy to use and set up. The placement of the components depends on the participant’s bio-metric characteristics in order to collect and extract accurate data during the experiments. For each participant the system has to be calibrated to the individual, as presented for each test in the following subsections. The test completion time is crucial, given that slower time than normal could be an indication of a medical condition. In all of the experiments the time of completion was measured.

### 4.1. TUG Experiments

**Figure 1:**
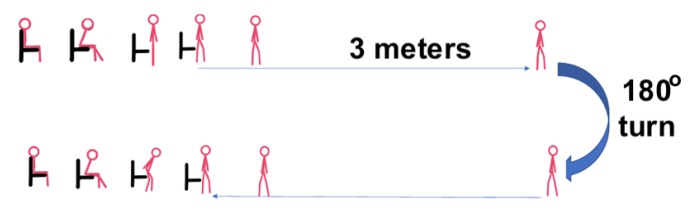
Timed up and go test experiment.

Inline with NHS instructions, subjects were seated on an armed chair, and on the word “Go”, the subjects would stand, walk 3 m on a straight line, make a 180°degree turn, walk back to the chair, turn and sit down (Figure 1).

**Figure 2:**
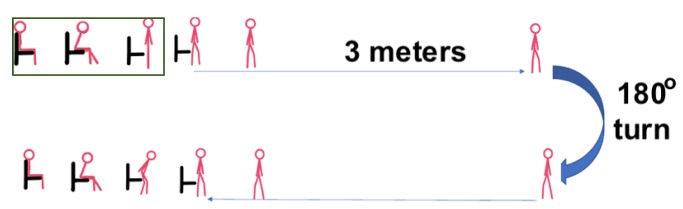
Timed up and go test experiment simulating difficulty to stand.

For calibration, each participant was asked to complete the TUG test at their normal walking speed (own pace) three times. Next, to investigate the properties of the system under a wide range of conditions, we asked the participants to simulate three impairments, and motion at an accelerated pace. First, they were asked to simulate reduced ability, or difficulty, to stand (Figure 2). The subject was trying to stand up by spending time on various positions or by performing unsuccessful attempts. This was a way of testing that the device and the motion sensor will be capturing and transmitting data accurately with the right angle and range without resetting and recapturing the particular subject multiple times. The task of sitting down was performed in a similar manner during this simulation.

**Figure 3:**
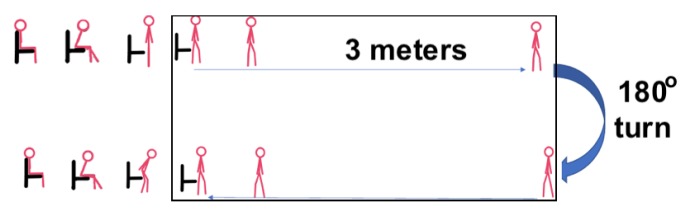
Timed up and go test experiment simulating difficulty to walk.

Next the participants were asked to simulate a reduced ability, or difficulty, to walk as it is often the case for patients with reduced mobility even if the distance is limited to 3 m (Figure 3). Subjects were asked to slow down in order to ensure that the time captured will be the time of the worst-case scenario (i.e., the time a geriatric elderly would need to perform this test). The aim was to evaluate the system’s ability to accurately capture the overall time, without system resets and without the sensor recording interference from the testing area.

**Figure 4:**
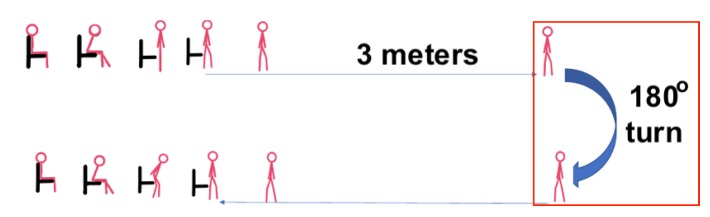
Timed up and go test experiment simulating difficulty to turn.

Finally, the participants were asked to simulate reduced ability or difficulty to turn by delaying when they were performing the 180° turn. This was simulated as wobbling or assuming the need of a walking aid (see Figure 4). Here, the subjects were asked to simulate imbalance while turning to capture the motion that are relevant to this stage.

This part of the experiment was designed to demonstrate the ability of the automated sensor system to recognise and capture potential mobility problems by distinguishing between these stages. There were no time restrictions for the participants to carry out and complete each of the five repetitions.

Finally, we tested the system under fast walking conditions. The participants were asked to perform the TUG test as fast as they could without running. The aim of this set was to identify the limitations of very low-cost sensors, in accurately capturing fast motion. The number of times each stage was repeated is presented in Table 2.

### 4.2. FTSTS Experiments

**Figure 5:**
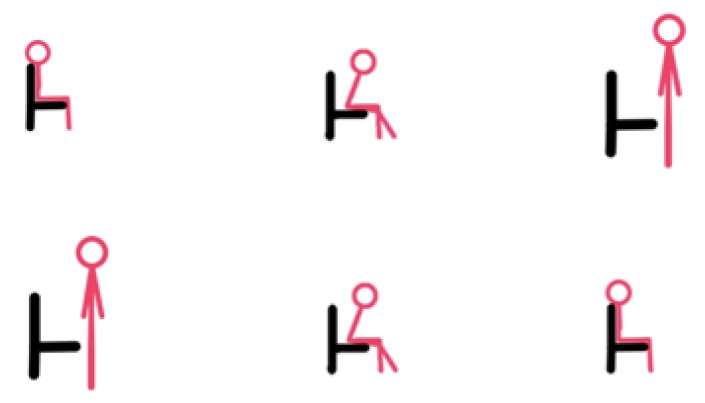
Five times sit to stand test stages repeated five times for each experiment.

Instructions were initially given to the participants on how to perform the FTSTS test. Each subject was seated on an armless chair with hands crossed over the chest. On the word “Go”, the participant had to stand and then sit five times without support (Figure 5). The participants performed experiments, at their own pace.

Next, the participants were asked to simulate difficulty to stand and sit, by performing the same task but with a delay in sitting and standing to validate if the sensor-system could accurately record these cases. Table 2 summarises the experiments.

### 4.3. Questionnaire

Each participant was asked to complete a questionnaire at the end of the experiment to identify the level of motivation and engagement. A total of five questions were provided with the opportunity to provide general comments at the end. The questions asked were:

1. Was the device easy to use and set up?
2. Was the feedback sufficient?
3. Would you use the device again?
4. Was the device engaging?
5. Did the device increase your motivation for performing the task?
6. Any other thoughts, detailed responses to the above questions, recommendations, or general comments?

The participants were asked to respond on a scale from 0, meaning very-poor, to 5 meaning excellent, to questions 1 to 5. The last question was open ended to allow for further feedback.

## 5. Results

Table 3 and Table 4 present the $\bar{\chi }$ and σ values for each of the TUG and FTSTS tests, respectively. The last column presents results from studies with adult patients reported in [53,54]. As demonstrated in the last column, the ranges of completion time recorded in the experiments were within the reported ranges for those patient populations. A two sample t-test analysis comparing the automated sensor system to the database supports the hypothesis that the two distributions are significantly similar for all cases; where α=0.05 is the acceptance limit. The t-values were −2.14, 1.42, 2.51, 0.27, 2.25, 6.66 and 0.98 for each case. The probability of the two distributions being equal was calculated to $0.99\geq P(|t|\geq t_{value})\geq 0.91$ for all other cases, except the fast TUG set with P(|t|≥0.273)=0.61 and the fast FTSTS set with P(|t|≥0.982)=0.83.

Table 5 presents the correlation of variation as a percentage for each set. To further investigate the correlation we use three different categories of graphs for each of the tests and each simulated impairment; box plots, linear regression, and Bland–Altman.

For the box plots, the *y*-axis represents the time while on the *x*-axis the methods of recording are shown. The whiskers which are lines anchored at the edges of the box, represent the range of measurements in seconds. The horizontal line in the box represents the mean and the *x* point in the box represents the median.

Bland–Altman plots, in our case, show the mean and the difference between video and system measurements, respectively, with acceptance limits within 2 s. The limits of agreement were calculated at 1.96×σ(differences) per definition for the 95% bias analysis [55,59]. The *y*-axis represents the difference between two measurements, i.e., video - system, while on the *x*-axis the mean of the measurements is shown. The horizontal black line represents the bias while the horizontal blue and red dotted lines represent the Upper and Lower level of agreement, respectively.

In the linear regression graphs, the *y*-axis represents the measurement of the system in seconds while the *x*-axis represents the video measurements in seconds.

The video recording was used as the “gold standard” or “ground truth" measurement for all the experiments to calculate the Lin’s coefficient, as well as the regression analysis.

**Figure 6:**
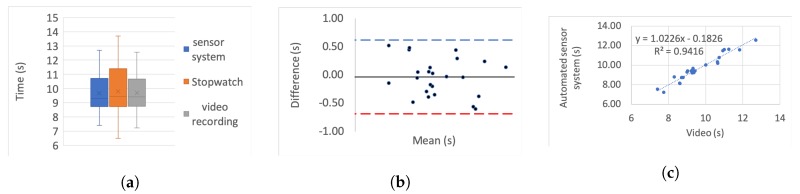
TUG normal simulation aggregate: (**a**) box plot; (**b**) Bland–Altman; (**c**) linear regression.

**Figure 7:**
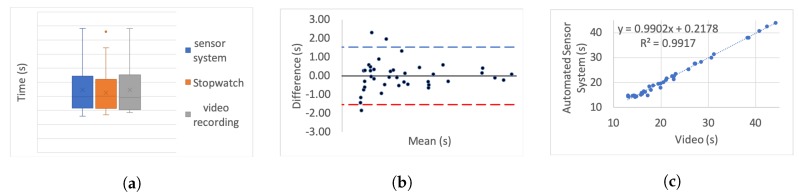
TUG difficult to stand simulation aggregate: (**a**) box plot; (**b**) Bland-Altman; (**c**) linear regression.

**Figure 8:**
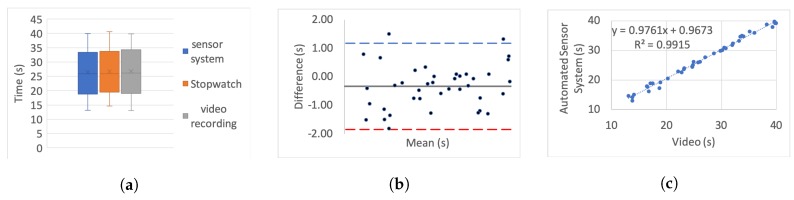
TUG difficult to turn simulation aggregate: (**a**) box plot; (**b**) Bland–Altman; (**c**) linear regression.

**Figure 9:**
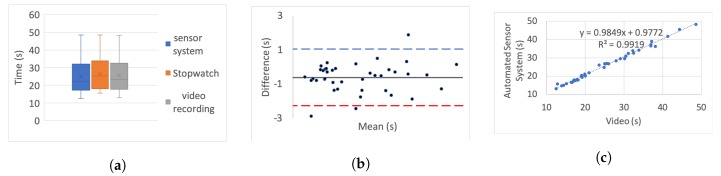
TUG difficult to walk simulation aggregate: (**a**) box plot; (**b**) Bland–Altman; (**c**) linear regression.

**Figure 10:**
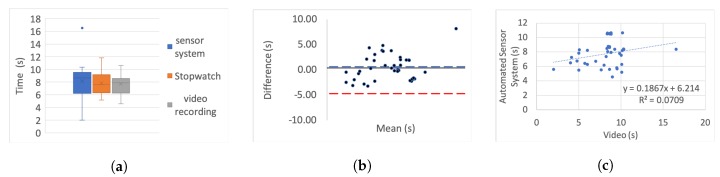
TUG fast simulation aggregate: (**a**) box plot; (**b**) Bland–Altman; (**c**) linear regression.

**Figure 11:**
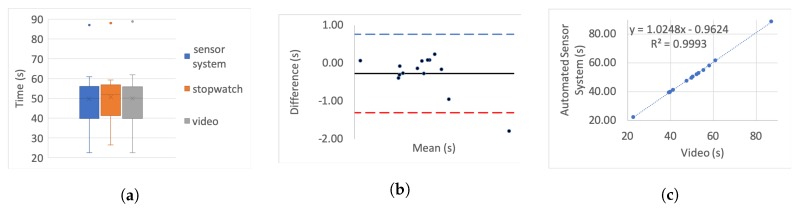
Five time sit to stand test slow simulation aggregate: (**a**) box plot; (**b**) Bland–Altman; (**c**) linear regression.

**Figure 12:**
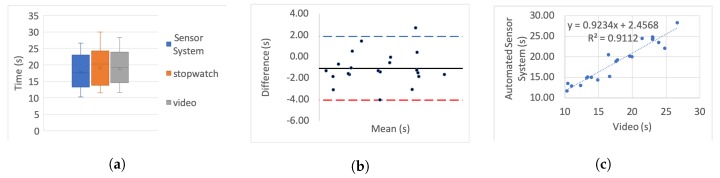
Five time sit to stand test fast simulation aggregate: (**a**) box plot; (**b**) Bland–Altman; (**c**) linear regression.

Figure 6, Figure 7, Figure 8, Figure 9, Figure 10, Figure 11 and Figure 12 present the results for TUG and FTSTS tests according to the simulated impairment or set of repetitions the participants were called to perform. Table 6 presents the PE, ϱc and ICC results of the analysis as defined in Section 3 and the linear regression results of the coefficient of determination ($R^{2}$) and *p*-value.

The responses from the collected questionnaires were analysed by simple sum and percentage proportion analysis for each of the possible responses. All of the subjects found the system easy to setup and use (Question 1: 100%≥3) meaning that all eight responses were equal to 3, 4 or 5. The 83% found the received feedback sufficient, good or excellent (Question 2: 83%≥3). Additionally, all the participants found the system engaging (Question 4: 100%=5) and that it increased their motivation to perform the task (Question 5: 100%=5). Finally, the majority would use the system again if they ever needed home self-rehabilitation (Question 3: 88%≥3).

## 6. Discussion

Although the task was the same for each of the impairments, as expected, the completion time of each participant was very different. Moreover, there was a significant difference between participants, as evidenced through the range of results presented in the box plot analysis. The automated sensor system has consistently produced box plots that are well aligned with the video recording. Thus further analysis was conducted to investigate correlation and agreement. The results of the simulated difficulties were hypothesised to represent adult participants with reduced mobility. The hypothesis was tested against the international database’s records for male and female patient groups. The result of the two-sample t-test hypothesis testing confirmed the validity of the assumption. As a result the device is suitable for recording TUG and FTSTS tests for a wide range of the patient population. It was further hypothesised that the system will demonstrate the same behaviour if used by female subjects. This hypothesis was supported by the comparative analysis to the international database where the adult groups had a strong female participant population (49.23% average of the studies that reported male/female ratios on TUG and FTSTS).

The stop-watch has lower correlation with the video as demonstrated from the box plots through displacement line. This difference is likely to be due to human error. Due to the significant variability in recording time, the stopwatch measurements were not regarded as the “ground truth” for the comparative analysis, and were not used for the Bland–Altman analysis. Additionally, the box plot analysis highlights the presence of outliers in the TUG fast series and the FTSTS simulated disability case.

The TUG fast series produced generally the poorest results as the participants were performing the test at a speed that exceeded the capabilities of the evaluated system. Thus the outlier in this case can be assigned to a system fault in capturing fast completion times. An outlier of the FTSTS test, on the other hand, is present in all of the three measurement methods and thus can be the result of a particular participant taking long pauses while performing the test.

For the Bland–Altman analysis all the data points (>50%) were between the limits of agreement. Points outside the limits of agreement are present in the TUG difficulty to stand simulation, turn simulation and the FTSTS fast test. However, in all cases these points are still very close to the LOA and do not statistically significantly affect the agreement between the two measurements. The bias was in most graphs close to zero, which reflects an unbiased relation between the measurements. It is worth noticing that in the graph which represents the TUG fast simulation, the upper limit of agreement coincides with the bias and it is obvious that the automated sensor system has limitations in recording fast repetitions.

By observing the linear regression analysis we can distinguish that, even in the worst case, i.e., the tests of TUG normal and FSTS fast simulation, the correlation is statistically significant. However, the TUG test fast simulation is completely uncorrelated, demonstrating that the automated sensor system is incapable of capturing fast movements. However, these recordings would be only relevant to adults who are potentially not in need of rehabilitation. The remaining TUG and FTSTS test sets are highly correlated with perfect alignment on the linear trend line. The above observations are further supported through the ICC, ϱc and $R^{2}$ results.

Finally, the responses to the questionnaire can be valued as an early indicator of engagement and motivation enhancement in adults. However, further experiments with an elderly population are necessary to draw any further conclusions.

## 7. Conclusions

The low cost, non-intrusive, non-wearable, motivation and engagement enhancing system that can be individualized and support daily activities, is cost-effective, non-complex and transferable has excellent correlation and agreement with the video recordings in all the simulated conditions. The stopwatch measurements have an inherently higher PE compared to the golden standard video measurements due to the human error factor. Moreover, the transferability of the automated sensor system is presented with the FTSTS test simulation demonstrating excellent accuracy and correlation to the video recording. The relevance of this early technology to the patient population was demonstrated through comparative analysis with the international database. However, experiments with elderly subjects will be required as further evaluation steps.

Fast FTSTS ($R^{2}=0.92$) was not as accurately captured while the fast TUG test was uncorrelated between system and video ($R^{2}=0.07$). The limitation of the very low-cost motion detection sensor is apparent in these two sets of experiments as the sensor’s delay in recording the event is significant and affects the recorded time. However, as the system is designed to be utilised for rehabilitation and incorporation of daily activities for increased engagement, the range of fast TUG is assumed with the scope of the study targeting less capable adult subjects. Thus, the automated sensor system is fit for purpose and has been validated for use with statistically significant accuracy (ϱc>0.99, $R^{2}>0.94$, ICC>0.96).

## Figures and Tables

**Table 1:** Medical tests to assess patient activities.

Test	Measured Capability
Functional reach test	Dynamic balance [10]
Berg Balance scale	Dynamic and static balance [11]
Performance oriented mobility assessment	Dynamic/static balance and gait abilities [12]
Balance evaluation system	Overall balance. Tests include sit to stand test, rise to toes, stand on one leg [13]
Falls efficacy scale and balance confidence scale self-evaluation	Subject ability/confidence in carrying out daily activities [14]
Balance error scoring system (BESS)	Static postural stability [15]
Timed up and go (TUG)	Mobility, static and dynamic balance [4]
Five times sit to stand (FTSTS) test	Lower-limb functionality, durability and balance [16]

**Table 2:** Timed up and go test and five time sit to stand experiments.

Test	Stage	Repetitions	Simulations
TUG	1	3	Normal walking
TUG	2	5	Difficulty to stand
TUG	3	5	Difficulty to walk
TUG	4	5	Difficulty to turn
TUG	5	5	Fast walking
FTSTS	1	3	Fast
FTSTS	2	2	Difficulty to stand and sit

**Table 3:** Timed up and go (TUG) characterisation of the tests: $\bar{\chi }\left(\sigma \right)$. The results are given in seconds.

TUG Set	Automated Sensor System	Stopwatch	Video	Patient [53]
Walk	24.94 (9.41)	26.46 (9.0)	25.54 (9.31)	31.9 (20.9) _(Geriatric age $\bar{\chi }$ 79.9)_
Turn	26.43 (8.25)	26.63 (7.92)	26.78 (8.1)	23.33 (11.66) _(Bilateral vestibular hypofunction age $\bar{\chi }$ 62.5)_
Stand	22.32 (8.65)	21.27 (7.01)	22.31 (8.6)	15.5 (11.03) _(Parkinson’s Fallers/No medication age $\bar{\chi }$ 77.95)_
Fast	8.13 (2.47)	7.82 (1.9)	7.73 (1.73)	7.94 (2.15) _(Parkinson’s Non Fallers/Medication age $\bar{\chi }$ 66.64)_
Normal	9.66 (1.30)	9.80 (1.8)	9.69 (1.37)	8.13 (2.34) _(Parkinson’s Non Fallers/No medication age $\bar{\chi }$ 66.64)_

**Table 4:** Five times sit to stand (FTSTS) characterisation of the tests: $\bar{\chi }\left(\sigma \right)$. The results are given in seconds.

FTSTS Set	Automated Sensor System	Stopwatch	Video	Patient [54]
Diff.	49.71 (14.58)	50.66 (14.05)	49.98 (14.94)	20.25 (14.12) _(Parkinson’s age $\bar{\chi }$ 65.9)_
Fast	17.78 (5.1)	19.15 (5.37)	18.87 (4.93)	16.4 (4.4) _(Vestibular disfunction age $\bar{\chi }$ 66.64)_

**Table 5:** Characterisation of the tests: coefficient of vitiation percentage (%).

Set	Automated Sensor System	Stopwatch	Video
TUG Walk	37.72	34.00	36.42
TUG Turn	31.23	29.75	30.23
TUG Stand	38.76	32.97	38.54
TUG Fast	30.46	24.33	22.45
TUG Normal	13.49	18.41	14.16
FTSTS Diff.	29.33	27.73	29.90
FTSTS Fast	28.69	28.08	26.14

**Table 6:** Characterisation of the tests: percentage error and correlation results.

Set	PE	ϱc	$R^{2}$	*p*-Value	ICC
TUG Walk	13.3%	0.994	0.992	0.058	0.994
TUG Turn	11.4%	0.996	0.992	0.022	0.995
TUG Stand	13.8%	0.996	0.992	0.924	0.996
TUG Fast	126.15%	0.246	0.079	0.00	0.251
TUG Normal	14.1%	0.997	0.942	0.140	0.969
FTSTS Diff.	4.73%	0.999	0.999	0.030	0.999
FTSTS Fast	31.1%	0.931	0.912	0.059	0.934
